# Population genetic structure is shaped by historical, geographic, and environmental factors in the leguminous shrub *Caragana microphylla* on the Inner Mongolia Plateau of China

**DOI:** 10.1186/s12870-017-1147-7

**Published:** 2017-11-13

**Authors:** Bo Xu, Guoli Sun, Xuemin Wang, Jingwei Lu, Ian J. Wang, Zan Wang

**Affiliations:** 1 Institute of Animal Sciences, Chinese Academy of Agricultural Sciences, Beijing, 100193 China; 2 Animal Science and Technology College, Jilin Agricultural University, Changchun, 130118 China; 3 Department of Environmental Science, Policy, and Management, University of California, Berkeley, CA 94720 USA

**Keywords:** Ecological niche, Isolation by distance, Isolation by environment, Plant ecology, Population genetic structure, Restoration ecology

## Abstract

**Background:**

Understanding how landscape factors, including suites of geographic and environmental variables, and both historical and contemporary ecological and evolutionary processes shape the distribution of genetic diversity is a primary goal of landscape and conservation genetics and may be particularly consequential for species involved in ecological restoration. In this study, we examine the factors that shape the distribution of genetic variation in a leguminous shrub (*Caragana microphylla*) important for restoration efforts on the Mongolian Plateau in China. This region houses several major bioclimatic gradients, and *C. microphylla* is an important restoration species because it stabilizes soils and prevents advancing desertification on the Inner Mongolia Plateau caused by ongoing climate change.

**Results:**

We assembled an expansive genomic dataset, consisting of 22 microsatellite loci, four cpDNA regions, and 5788 genome-wide SNPs from ten populations of *C. microphylla*. We then applied ecological niche modelling and linear and non-linear regression techniques to investigate the historical and contemporary forces that explain patterns of genetic diversity and population structure in *C. microphylla* on the Inner Mongolia Plateau. We found strong evidence that both geographic and environmental heterogeneity contribute to genetic differentiation and that the spatial distribution of genetic diversity in *C. microphylla* appears to result partly from the presence of a glacial refugium at the southwestern edge of its current range.

**Conclusions:**

These results suggest that geographic, environmental, and historical factors have all contributed to spatial genetic variation in this ecologically important species. These results should guide restoration plans to sustain genetic diversity during plant translocations.

**Electronic supplementary material:**

The online version of this article (10.1186/s12870-017-1147-7) contains supplementary material, which is available to authorized users.

## Background

Unraveling the factors that influence spatial genetic variation and population structure is one of the fundamental goals of ecological and landscape genetics [1]. Patterns of genetic differentiation often reflect spatial variation in gene flow, and landscapes can influence gene flow through geographic and environmental variation and their combined effects [2–4]. Isolation-by-distance (IBD) is the correlation of genetic divergence and geographic distances, while isolation-by-environment (IBE) is a correlation between genetic divergence and environmental dissimilarity [5, 6]. IBE can result from environmental differences between populations that generate divergent selection, which reduces dispersal success between different environments, or from biased dispersal, which leads to higher dispersal rates between more similar environments [2, 3, 6, 7]. Thus, both IBD and IBE represent important ways in which landscape heterogeneity influences genetic structure in natural populations [3, 8, 9]. Inherently, geographic and environmental isolation are not mutually exclusive, and spatial genetic divergence among populations can result from reduced gene flow associated with both geographical and ecologic factors [2, 7, 8, 10]. The rise of modern spatial statistical methods and the increasing availability of high-resolution geographic and environmental data layers now make it possible to accurately describe geographic and ecological landscapes and to simultaneously estimate the effects of IBD and IBE on spatial genetic divergence [5, 6]. Understanding patterns of IBD and IBE is particularly important for species of conservation concern or that are involved in ecosystem management, because the outcomes of conservation strategies may depend upon properly managing genetic diversity.

One such species is *Caragana microphylla*, a perennial sandy grassland and desert deciduous shrub species belonging to the legume family (Fabaceae). Native to temperate Asia, including Siberia, Mongolia, and China [11], *C. microphylla* is a widely distributed shrub species in the northern steppe and agro-pastoral ecotone of China. On the high plain of the Inner Mongolia Plateau, *C. microphylla* is a key component of the shrub steppe landscape, and on the sandy land of the steppe it is a dominant species of vegetation [11]. The species has been valued for its tolerance to heat, cold, and drought and for its resistance to wind erosion, sand burial, and hail storms. It has been used as a pioneer leguminous shrub species for vegetation rehabilitation and stabilization of widely degraded and degrading grasslands in China, because of its ability to serve as a windbreak and its capacity for carbon fixation, nitrogen fixation, and nutrient accumulation in sandy soils [12], and it can also be served as supplemental livestock forage with high nutrient value [12]. Genetic variation and population structure of wild *C. microphylla* from the Inner Mongolia Plateau have been evaluated by different marker systems, including AFLPs, RAPDs, and microsatellites [13–17], but no previous studies have quantified the contributions of IBD and IBE to spatial genetic divergence in this system. However, better understanding population dynamics in species like this is an important goal for restoration ecology, ecosystem management, and landscape and conservation genetics. Studies like this one are critical for identifying the factors that shape the distribution of genetic variation in species undergoing assisted dispersal and recolonization so that genetic diversity can be managed properly [18].

The Inner Mongolia Plateau is characterized by pronounced biophysical gradients, presenting an opportunity to investigate the effects of multiple geographic and environmental factors on population connectivity. A temperature gradient runs roughly North-South, while a precipitation gradient runs from arid regions in the Southwest to wetter regions in the Northeast. An ecotone largely tracks the precipitation gradient, transitioning from desert in the Southwest to grassland (high meadow and steppe) in the central plateau and forest in the Northeast, with pockets of shrublands and sandy lands in the Southeast. Here, we evaluated population genetic divergence of *C. microphylla* across its entire geographic range on the Inner Mongolia Plateau, using a set of 22 polymorphic microsatellite markers, four cpDNA sequences and 5788 SNPs generated through genotyping-by-sequencing (GBS). The major goals of this study were (i) to characterize genetic variation and population structure in *C. microphylla*, and (ii) to quantify the relative contributions of geographic factors (IBD) and environmental clines (IBE) to genetic differentiation in this important restoration species.

## Methods

### Population sampling and DNA isolation

**Fig. 1:**
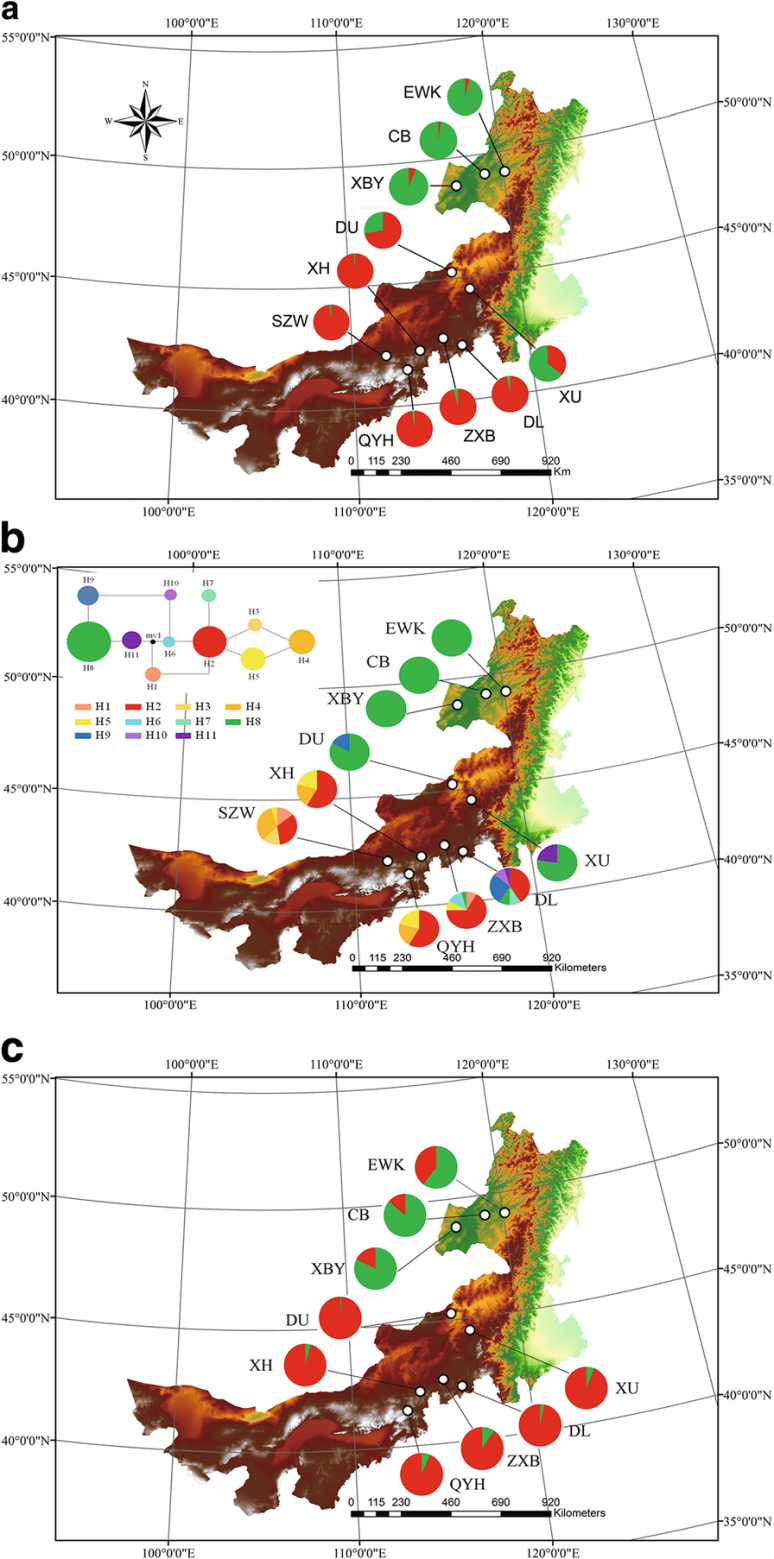
Population locations for the 10 sites sampled in our study and their associated genetic diversity. The pie charts next to each population indicate their proportions of assignment to two genetic clusters based on Structure analysis, for our microsatellite (**a**) and GBS (**c**) datasets, or their haplotype diversity, for our cpDNA dataset (**b**). Panel B also includes a haplotype network in which the sizes of the colored circles are proportional to the frequencies of the they represent

We collected samples from 221 individuals of *C. microphylla* at ten sites throughout the natural distribution of the species in the southern Inner Mongolia Plateau of China (SZW, ZXB, DL, XH, and QYH), central Inner Mongolia Plateau (XU and DU), and northeastern Inner Mongolia Plateau (EWK, CB, XBY) (Additional file 1: Table S1 and Fig. 1). Together, these sites cover a wide range of climate space, giving us good power to detect environmentally-driven spatial genetic variation (Additional file 1: Figure S1). Sample sizes ranged from *N* = 18 to 24, with a mean of *N* = 22 (Additional file 1: Table S1). Total genomic DNA was extracted from leaf tissues using the Qiagen DNeasy Plant Kit, according to the manufacturer’s protocol (Qiagen, Hilden, Germany).

### Microsatellite genotyping

We genotyped all samples at 22 microsatellite (simple sequence repeat; SSR) markers (Additional file 1: Table S2) that were developed by Han [19] using the method described by Lian et al. [20] (Additional file 1: Table S2). PCR amplification was conducted in a total volume of 25 μL including 40 ng DNA, 1 × buffer, 3 mM MgCl_2_, 300 μM dNTPs, 0.6 μM forward primer and reverse primer, and 1 U Taq DNA polymerase (TaKaRa, Shiga, Japan). The forward primers were tagged with a fluorescent 6-FAM or HEX label to produce flourescent-labeled PCR amplified fragments. PCR was performed on a Mastercyler gradient thermocycler (Eppendorf, Hamburg, Germany) using the following procedure: 10 min at 94 °C, followed by 10 touchdown cycles of 45 s at 94 °C, 60 s at 65 °C (−1 °C per cycle), and 60 s at 72 °C, then 35 cycles of 45 s at 94 °C, 60 s at 55 °C, and 60 s at 72 °C, and a 10 min final extension step at 72 °C. An ABI3730xl DNA Analyzer (Applied Biosystems, Foster City, CA, USA) was used to capture amplified products by a fluorescence detection system for SSR markers. Fragment sizes were determined using an internal size standard (LIZ500, ABI, USA), and the output was analyzed using GeneMapper software (Applied Biosystems).

### *cpDNA* sequencing

Four cpDNA regions, including *trnL-trnF*, *psbA-trnH*, *psbB-psbH* and *trnG,* were amplified for all 221 individuals. The primers and methodology for amplification of these four DNA regions via PCR were described in Taberlet et al. [21], Demesure et al. [22], Hamilton [23], and Shaw et al. [24], respectively. Sequences were generated with an ABI 3730XL DNA Sequencer (Applied Biosystems), and edited, assembled and aligned in Geneious (v7.1.7, http://www.geneious.com/). All cpDNA sequences were deposited in Genbank (accession numbers KU564257 to KU564268).

### GBS sequencing, data filtering and genotyping

A total of 127 samples from ten populations were used to generate the genotyping by sequencing dataset (GBSseq; Additional file 1: Table S1). Individual DNA libraries for each of these samples were prepared using the restriction enzyme *ApeK*I according to the protocol in Elshire et al. [25]. Libraries were then sequenced using paired-end sequencing across 3 lanes of Illumina HiSeq 4000 (BGI, Shengzhen, China). The quality of the raw read data was examined using FASTQC [26]. GBS data assembly, mapping, and SNP discovery were performed using Stacks v1.23 [27]. In the absence of a reference genome for this species, RADSeq loci were assembled de novo using the ‘denovo_map.pl’ pipeline in STACKS. We used a parameter combination recommended by Mastretta-Yanes et al. [28]: minimum read depth to create a stack (−m) = 3, number of mismatches allowed between loci within individuals (−M) = 2, and number of mismatches allowed between loci within each catalogue (−n) = 2. All other parameters were kept at default values. Those loci present in at least 80% of individuals at each site were retained in the final dataset, and loci with minor allele frequencies lower than 0.05 were removed. GBS genotyping of the samples from population SZW resulted in significant missing data, so this population was removed from the GBS dataset. GBS-seq raw data were submitted to the NCBI Sequence Read Archive (SRA) with reference number SRP071628.

### Microsatellite analysis

All 22 microsatellite loci were tested for deviation from Hardy-Weinberg equilibrium. We calculated common metrics of genetic variation, including average number of alleles (Na), observed and expected heterozygosity (Ho and He), and global and pairwise F_ST_. Both global and pairwise F_ST_ were tested for significance based on 9999 permutations. All calculations and statistical tests were conducted using GenAlEx v6.5 [29].

We used the software STRUCTURE to infer the probability of assignment to distinct genetic clusters for all 221 individuals in the ten sampled populations [30]. The analysis was performed using the admixture model and with the option of correlated allele frequencies between populations. Ten runs were conducted for each value for the number of genetic clusters (K), with K ranging from 1 to 10. The length of the burn-in for the Markov Chain Monte Carlo (MCMC) replications was set to 10,000, and data were collected every 1000 steps over a total length 100,000 MCMC steps in each run. We identified the optimal value of K using the method developed by Evanno et al. [31] as implemented in the software Structure Harvester [32].

### cpDNA analysis

Chloroplast DNA (cpDNA) sequences were edited and assembled using SeqMan software (DNASTAR, Inc., Madison, Wisconsin, USA). Multiple alignments of the DNA sequences were performed with Clustal X [33], with subsequent adjustment in Bioedit [34]. Haplotype (*Hd*) and nucleotide (π) diversities were calculated from aligned DNA sequences using DnaSP v5 [35]. We conducted an analysis of molecular variance (AMOVA) and tested for significance based on 1023 permutations in Arlequin v3.0 [36]. A haplotype distribution map was constructed using ArcMap v9.3 (ESRI, Inc., Redlands, California, USA), and a haplotype network was constructed in NETWORK v4.678 [37] using *Medicago sativa* as an outgroup.

### GBS data analysis

Observed and expected heterozygosities (Ho and He) were calculated using the package Adegenet v2.0.1. [38] in R (www.r-project.org). Both global and pairwise F_ST_ were calculated and tested for significance based on 9999 permutations using Genepop v4.0 [39]. As with the microsatellite dataset, we also performed STRUCTURE analysis [30] on our GBS dataset, using the admixture model and the same MCMC parameters as before.

### Ecological niche modelling (ENM)

We used climate-based ecological niche models (ENMs) at multiple time periods to investigate whether current and past climate suitability is a relevant factor shaping observed patterns of genetic differentiation among populations of *C. microphylla*. Ecological niche modelling was carried out in MAXENT v3.3.3 [40]. A total of 53 occurrence points, obtained from the literature [13–16] and our own sampling, and 19 GIS data layers at 2.5 arc min resolution for present-day bioclimatic variables, obtained from the WorldClim database (http://www.worldclim.org), were included in the analysis (Additional file 1: Table S3).

To estimate the distribution of *C. microphylla* at the Last Glacial Maximum (LGM), we projected the model obtained by our present-day species-climate analysis onto the LGM using data layers for past climate constructed under the commonly used Model for Interdisciplinary Research on Climate (MIROC v3.2) [41] scaled down to a 2.5 arc min resolution and obtained from WorldClim. To model the suitability of *C. microphylla* under a future climate scenario, we acquired data layers (2.5 arc min resolution) predicted for the year 2080 under, again, the commonly used general circulation model MIROC from WorldClim. As with the LGM scenario, we projected the present-day ENM onto the future climate layers to explore how the predicted distribution of *C. microphylla* may be affected by ongoing global climate change. The performance of the model prediction was evaluated using the area under the (receiver operating characteristic) curve (AUC) calculated by MAXENT. Model predictions were visualized in ARCMAP v9.3 (ESRI, Redlands, CA).

### Isolation by distance (IBD) and isolation by environment (IBE)

To investigate the roles of geographic and environmental factors in spatial genetic differentiation, we tested for isolation by distance (IBD) and isolation by environment (IBE) using all three marker types collected in our study. In all analyses, population pairwise genetic distances were represented by matrices of pairwise F_ST_ / (1-F_ST_) as recommended by Rousset [42]. Geographic distances were represented by the logarithms (log_10_) of geographic distances between all pairs of populations. For the environmental predictors, we downloaded the 19 bioclimate variables from the WorldClim database (www.worldclim.org) at 0.5 arc min resolution and then reduced covariance by performing spatial principal component analysis (PCA) analysis on the raster layers using the R package ‘RStoolbox’ [43]. We retained the first three PC rasters that resulted from this analysis and extracted the value of the data point on each raster at each of the coordinates for our sampled populations. To quantify IBD and IBE, we performed multiple matrix regression with randomization (MMRR) using the R function ‘MMRR’ [44]. We also tested for covariance between geographic and environmental distances with a Mantel test using the R package ‘vegan’ [45]. In each analysis, 10,000 permutations were used to generate a null distribution for significance testing.

To further evaluate IBD and IBE, we also performed a complementary analysis, generalized dissimilarity modelling (GDM). GDM is a statistical technique that uses nonlinear matrix regression to model spatial patterns of biological dissimilarity, including genetic distance, between sampling sites against differences in geographic and environmental variables [46]. GDM uses an I-spline turnover function for each predictor variable to quantify: (1) variation in the rate of genetic turnover along each environmental and geographic axis (the shape of each spline) while controlling for all other variables, and (2) the curvilinear relationships between genetic distance and geographic and environmental distances [46, 47]. The maximum height of each spline corresponds to the relative importance of the associated predictor [46].

Genetic distances for each model were the same F_ST_ / (1-F_ST_) matrices used in the MMRR analysis, and geographic distances were based on the geographic coordinates of each sampling site. For the environmental predictors, we used the same three PC rasters as in the MMRR analysis. After fitting the GDM model to these data, we visualized spatial patterns of genetic turnover by projecting the GDM model onto the PC rasters. This assigns a color value to each cell on the raster based on its predicted genetic composition, and greater differences in the colors between cells indicate greater predicted genetic differences. All of the GDM analyses were performed in the R package ‘gdm’ [48].

## Results

### Genetic variation of *C. microphylla*

Twenty-two microsatellite markers were used to evaluate genetic diversity across 221 individuals of 10 populations of *C. microphylla* (Table 1). The mean number of alleles per locus (Na) ranged from 5.318 in population XBY to 8.091 in population DU (Table 1). Mean observed heterozygosity per population (Ho) ranged from 0.416 in CB to 0.693 in QYH, and mean expected heterozygosity (He) values ranged from 0.490 in XBY to 0.708 in DU. We did not detect significant deviations from HWE in any of the 22 loci.

**Table 1:** Summary of genetic variation in *C. microphylla* populations detected in microsatellites (SSR), chloroplast DNA sequences (cpDNA), and genotyping-by-sequencing (GBS)

Population	SSR			cpDNA				GBS	
	Na	Ho	He	Ha	*Hd*	*π* × 10^3^		Ho	He
SZW	7.73	0.63	0.65	5	0.771	0.47		–	–
ZXB	8.00	0.61	0.67	6	0.554	0. 35		0.280	0.307
DL	7.41	0.58	0.64	6	0.766	0. 86		0.262	0.289
XH	7.55	0.65	0.66	3	0.598	0. 32		0.303	0.308
QYH	6.86	0.69	0.66	3	0.692	0.35		0.268	0.256
XU	7.18	0.46	0.58	2	0.368	0. 27		0.228	0.287
DU	8.09	0.68	0.71	2	0.294	0. 11		0.269	0.281
EWK	6.73	0.62	0.62	1	0.000	0. 00		0.261	0.261
CB	6.32	0.42	0.53	1	0.000	0. 00		0.196	0.247
XBY	5.32	0.43	0.49	1	0.000	0. 00		0.219	0.250

Na, the average number alleles per locus; Ho, observed heterozygosity; He, expected heterozygosity; Ha, number of haplotypes; *Hd,* haplotype diversity; *π,* nucleotide diversity

A total of 11 different cpDNA haplotypes (H1-H11) were identified based on 7 polymorphic sites detected in four cpDNA sequences (Table 1). Haplotypes H1 and H2 were the two most common haplotypes, found in 70% and 50% of *C. microphylla* populations, respectively (Fig. 1b). Haplotypes H3, H6, and H10, on the other hand, were found in only one population each (Fig. 1b). Haplotypes H8 and H4 were identified as the most ancestral and youngest haplotypes, respectively. The populations ETW, CB, and XBY had the lowest haplotype diversity (Hd) and nucleotide diversity (*π*) with only one haplotype (H8) observed in each population. The highest diversity was observed in population SZW (*Hd* = 0.771 and *π* = 0.47; Table 1).

Overall, 5788 SNPs from our GBS reads were retained for 106 individuals from 9 populations after quality control and filtering steps. Mean observed heterozygosity (Ho) per population ranged from 0.196 in CB to 0.303 in XH, and mean expected heterozygosity (He) values ranged from 0.247in CB to 0.308 in XH (Table 1).

### Population structure and genetic differentiation

Global F_ST_ among all 10 sampling sites based on our microsatellite dataset was 0.115. The pairwise estimates of genetic differentiation (F_ST_) across all 10 populations ranged from 0.017 (ZXB vs. XH) to 0.139 (XBY vs. QYH) (Additional file 1: Table S4). All *C. microphylla* population pairs were significantly differentiated from each other except for the ZXB and XH pair, for which F_ST_ was not significantly different from zero. Comparisons between regions revealed little structure between the central (DU and XU) and northeast (CB, EWK, and XBY) populations (mean F_ST_ = 0.071) nor between the central and southwest (QYH, SZW, DL, XH, and ZXB) populations (mean F_ST_ = 0.073), but did reveal higher differentiation between the northeast and the southwest populations (mean F_ST_ = 0.138). The population XBY showed the highest degree of genetic differentiation from other populations (mean F_ST_ = 0.171), followed by populations CB (mean F_ST_ = 0.168) and EWK (mean F_ST_ = 0.138).

For cpDNA, global F_ST_ among all sites was 0.360. About 76% of pairwise F_ST_ estimates among *C. microphylla* population pairs were statistically significant (Additional file 1: Table S5). Pairwise comparisons between regions revealed similar patterns compared to the microsatellite results. The mean genetic differentiation between northeast and southwest populations was F_ST_ = 0.64, followed by southwest vs. central populations (mean F_ST_ = 0.48), and northeast vs. central populations (mean F_ST_ = 0.17).

For our GBS dataset of 5788 SNPs, global F_ST_ among the nine sampling sites retained in this dataset was 0.246. Pairwise comparisons between regions revealed similar patterns compared to the microsatellite and cpDNA results (Additional file 1: Table S6). The mean genetic differentiation estimates between southwest vs. central populations and northeast vs. central populations were F_ST_ = 0.164 and F_ST_ = 0.168, respectively. Whereas, the mean genetic differentiation between northeast and southwest populations was F_ST_ = 0.350.

**Fig. 2:**
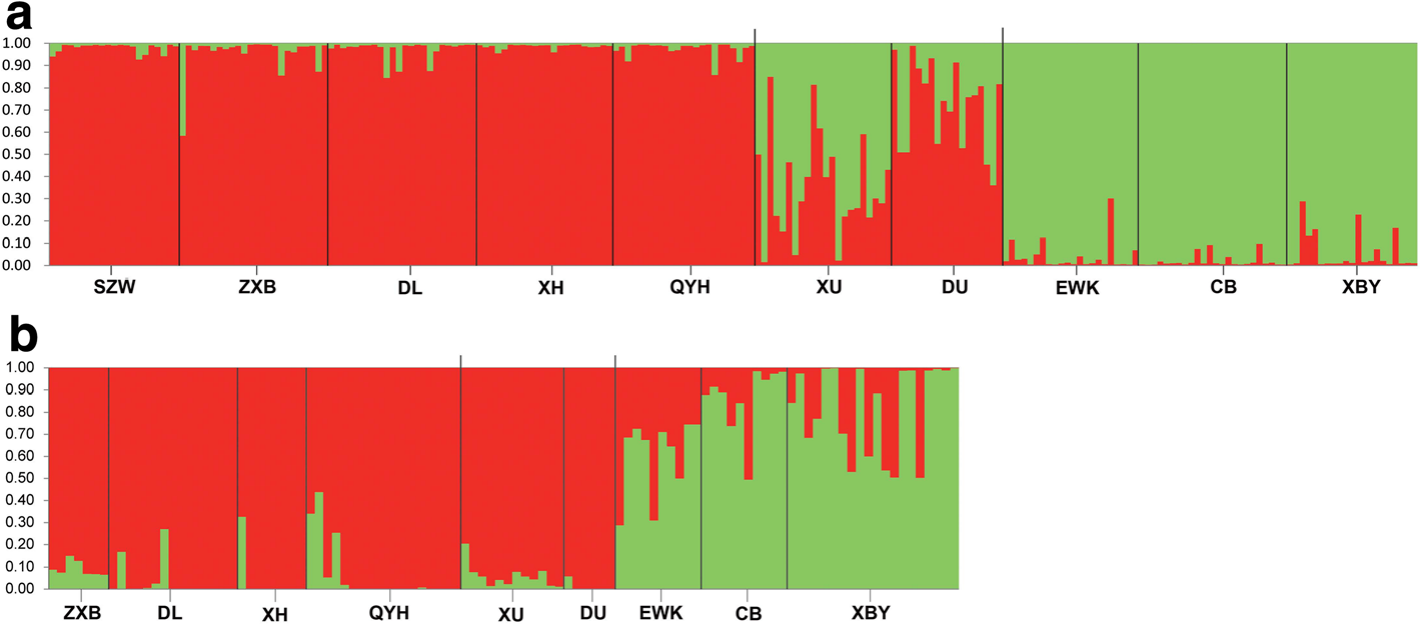
Results of Structure analysis for our microsatellite (**a**) and GBS (**b**) datasets. In each panel, each vertical bar represents the probabilities of assignment to two distinct genetic clusters for each individual. Individuals are grouped into the populations from which they were sampled

STRUCTURE analyses performed on both our microsatellite and GBS datasets indicated that the best supported number of clusters was K = 2, according to the ΔK methods for identifying the optimal number of clusters (Additional file 1: Figure S2). The probability of membership to either of the two clusters (A and B) was geographically structured among populations and regions (Figs. 1, 2). Specifically, the membership proportions in the STRUCTURE analysis revealed a significant geographic pattern in which individuals in populations mostly associated with cluster A (i.e. SZW, ZXB, XH, DL, and QYH) were more common in the southwest of the Inner Mongolia Plateau (41°N - 42°N; Fig. 1a, 2a), while individuals in populations mostly associated with cluster B (i.e. EWK, CB, and XBY) were found in the northeast of the Inner Mongolia Plateau (48°N- 49°N; Fig. 1a, 2a). In the central region of the Inner Mongolia Plateau (44°N - 45°N), two populations (XU and DU) showed intermediate probabilities of assignment to either cluster, based on the microsatellite dataset (Fig. 1a, 2a). This could result from potential admixture, shared ancestry, or demographic factors. Of the two central populations, although the XU population is more southerly, it shared genetic cluster assignments (35.6% in cluster A and 64.4% in cluster B) more with the populations in the northeast of the Inner Mongolia Plateau than with those in the southwest. The DU population, on the other hand, showed more similar genetic assignments (72.3% in cluster A and 27.7% in cluster B) to the populations in the southwest of the Inner Mongolia Plateau, even though it is geographically closer to the northeast populations (Fig. 1a, 2a). In contrast, the assignment probabilities for individuals in these populations based on the GBS dataset (Fig. 1c, 2b) were much more closely aligned with cluster A (the southwest populations) compared to those based on the microsatellite data (Fig. 1a vs. 1c; Fig. 2a vs. 2b).

### Ecological niche modelling of *C. microphylla*

**Fig. 3:**
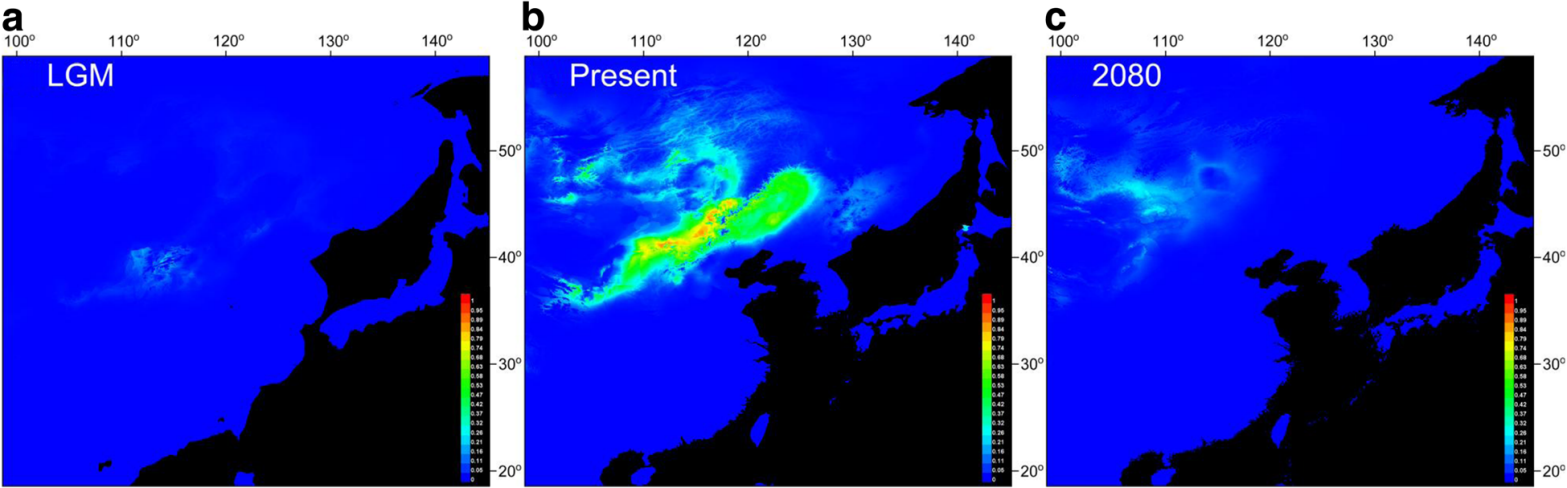
Predicted distributions of *C. microphylla* in China (**a**) at the Last Glacial Maximum (LGM; c. 21kya), (**b**) at present (1950–2000), and (**c**) in the future (2080). Each panel represents the probability of occurrence of *C. microphylla* in each cell on the map based on ecological niche modelling analysis

Three climate-based ecological niche models were constructed using 19 bioclimatic variables for three time periods: present day, the last glacial maximum (LGM), and the future (year 2080) (Fig. 3). The model based on present-day data showed strong support based on the receiver operating curve (AUC > 0.95), suggesting good model fit to the underlying data. The present-day predicted distribution of *C. microphylla* is consistent with its presently observed distribution (Fig. 3b). In total, precipitation had a greater influence on *C. microphylla* than temperature, as indicated by jackknife resampling of the regularized training gain (Additional file 1: Figure S3). Compared with its current distribution, the estimated distribution of *C. microphylla* during the LGM was much smaller, based on projection of the present-day model onto past climate layers, suggesting that a significant expansion occurred after the LGM from the southwest to the northeast of the Inner Mongolia Plateau (Fig. 3a). The ENM projected onto the future climate scenario for 2080 suggests that climate change will result in a significant reduction of the species’ potential range (Fig. 3c), resulting in a retraction to a small zone of climatically suitable habitat in the southwest-central part of *C. microphylla*’s current distribution.

### IBD and IBE

The spatial PCA that we performed on the 19 bioclimate data layers returned three PC rasters that explained >85% of the total bioclimatic variation. Factors loadings showed that PC1 was primarily described by temperature variables (Bio1–11; www.worldclim.org), while PC2 was derived from precipitation variables (Bio12–19; www.worldclim.org). PC3 was driven by three variables – precipitation seasonality (Bio15), mean diurnal temperature range (Bio2), and temperature annual range (Bio7) – and therefore represents an environmental seasonality and range of variation axis.

**Fig. 4:**
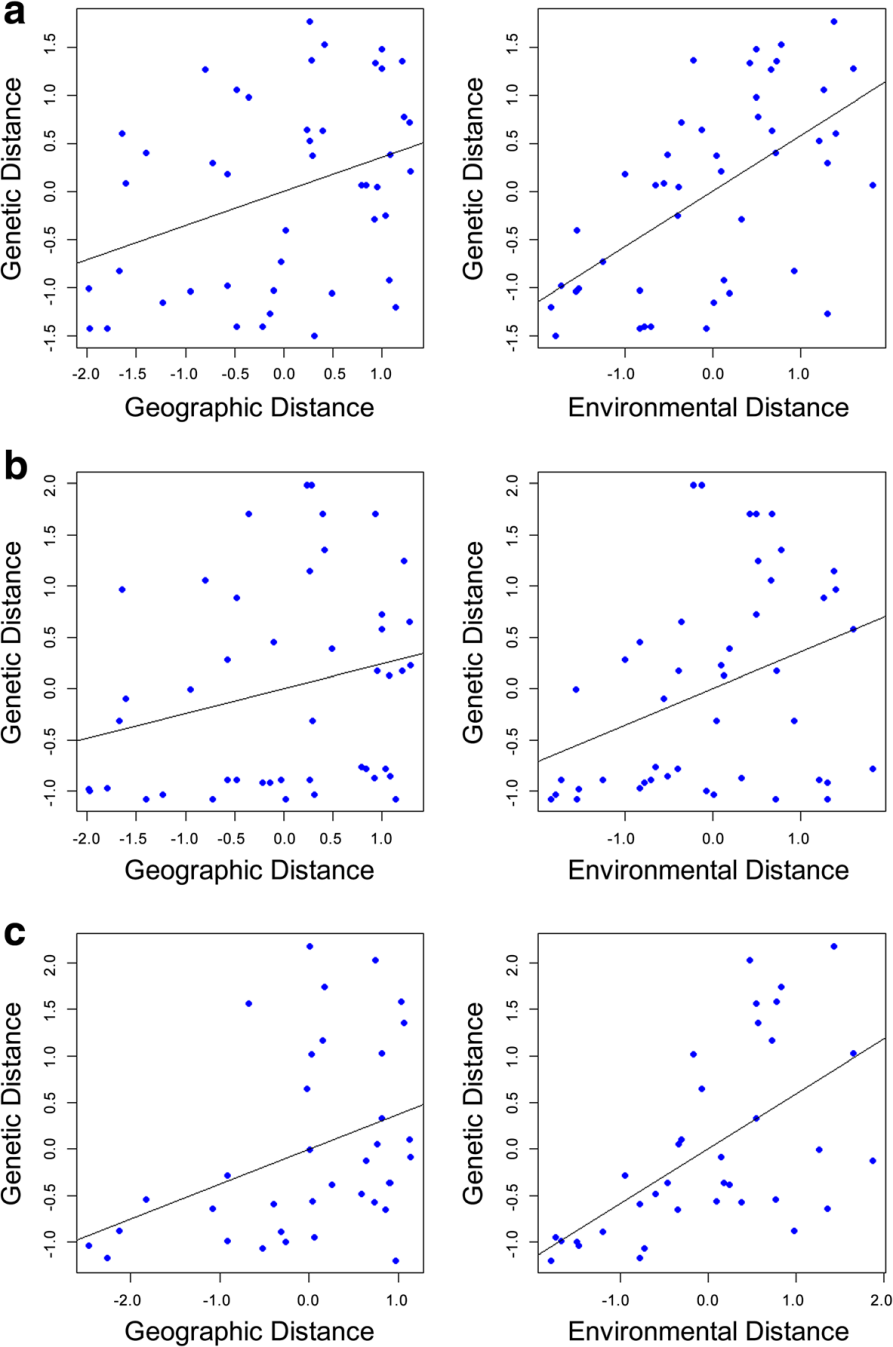
Scatterplots of genetic distance vs. geographic (left) and environmental distances (right) for each of our molecular datasets: microsatellites (**a**), cpDNA (**b**), and GBS (**c**). Each panel includes a simple, univariate regression line

Multiple matrix regression with randomization (MMRR) analysis suggested that genetic differentiation showed a significant pattern of both IBE and IBD for all three molecular datasets (Table 2 and Fig. 4). For each of the microsatellite, cpDNA, and GBS datasets, the model was a significant fit to the data (*p* < 0.001for each; Table 2), and explained a large proportion of the total variance (R^2^ = 0.685, R^2^ = 0.696, R^2^ = 888, respectively; Table 2). The signal of IBE in each dataset was driven by PC1 (temperature variables), which had a significant association with genetic distances in all three cases (*p* < 0.01 for each model; Table 2). PC2 and PC3 did not have significant correlations with genetic distances for any of the three molecular markers (*p* > 0.05; Table 2). IBE was slightly stronger than IBD in the cpDNA (IBE = 0.515, IBD = 0.361) and GBS datasets (IBE = 0.564, IBD = 0.444) and was considerably stronger than IBD in the microsatellite dataset (IBE = 0.702, IBD = 0.260; Table 2). Geographic distances were moderately correlated with distances in PC1 (Mantel’s *r* = 0.685, *p* = 0.001) and PC3 (Mantel’s *r* = 0702, *p* = 0.002) but showed no correlation with PC2 (Mantel’s *r* = 0.044, *p* = 0.553).

**Table 2:** Results of Multiple Matrix Regression with Randomization (MMRR) analysis for each of our three molecular datasets

	Model		IBD		PC1		PC2		PC3		IBE	
	R^2^	*p*	β	*p*	β	*p*	β	*p*	β	*p*	β	*p*
SSR	**0.685**	<0.01	**0.260**	<0.01	**0.549**	<0.01	0.006	0.94	0.159	0.31	**0.702**	<0.01
cpDNA	**0.696**	<0.01	**0.361**	0.02	**0.383**	<0.01	0.080	0.30	0.211	0.08	**0.515**	<0.01
GBS	**0.888**	<0.01	**0.444**	<0.01	**0.563**	<0.01	0.059	0.36	0.060	0.50	**0.564**	<0.01

The overall model fit (R^2^) and significance (*p*), regression coefficients (β) and *p*-values for each predictor variable (geographic distance [IBD] and environmental PCs [PC1, PC2, and PC3]), and cumulative coefficient of IBE (for all PCs) are shown. Significant values are in bold

**Fig. 5:**
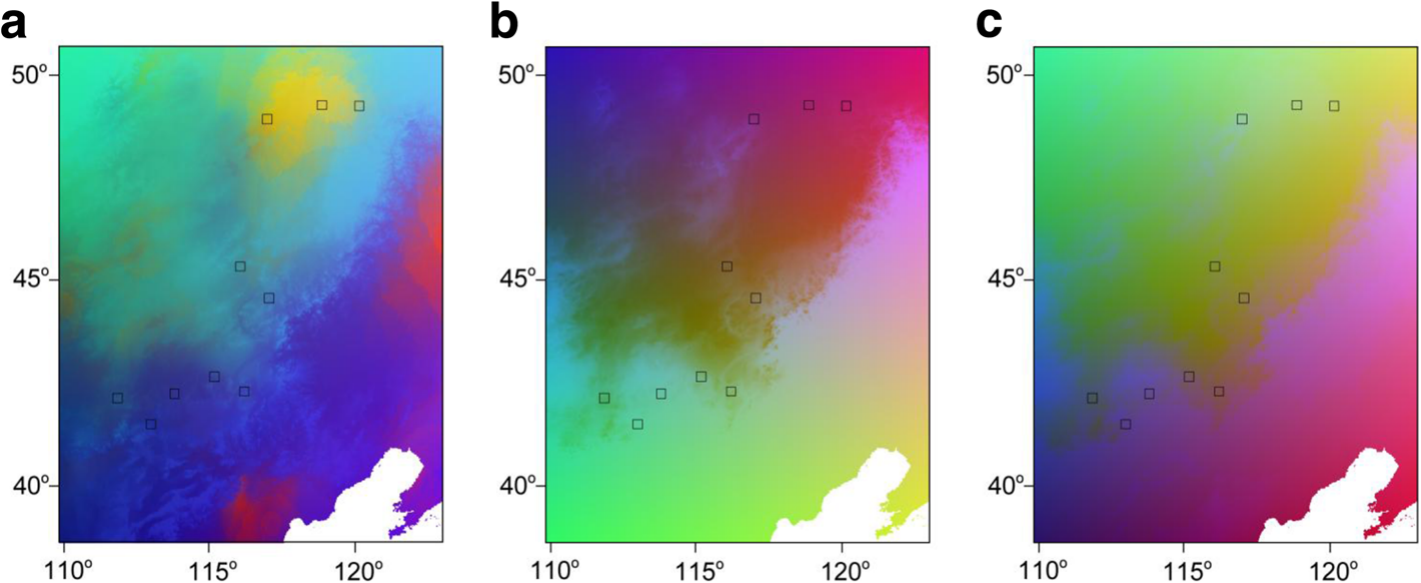
Predicted spatial genetic turnover in *C. microphylla*, based on generalized dissimilarity modelling (GDM) analysis for each of our molecular datasets: microsatellites (**a**), cpDNA (**b**), and GBS (**c**). The color of each cell on the map reflects its predicted genetic composition, and greater differences in the colors between cells indicate greater predicted genetic differences. Squares represent our sampling localities

Generalized dissimilarity modelling (GDM; Ferrier et al. 2007) analysis also revealed significant patterns of IBE and IBD in all three of our molecular datasets (Table 3). Overall, the models were a significant fit to the data (*p* < 0.01 for each model) and explained a large proportion of the deviance in the data structure, with 77.95% of deviance explained for the microsatellite dataset, 74.81% of deviance explained for the cpDNA dataset, and 89.18% of deviance explained for the GBS dataset. Deviance explained values for non-linear models are analogous to R^2^ values for linear models. Concordant with the results of the MMRR analysis, GDM revealed significant associations between genetic distances and PC3 distances for each dataset (*p* < 0.05; Table 3). IBE was stronger than IBD in the microsatellite dataset (IBE = 0.561, IBD = 0.132; Table 3) but slightly weaker than IBD in the cpDNA (IBE = 0.473, IBD = 0.627; Table 3) and GBS (IBE = 0.622, IBD = 0.717; Table 3) datasets. In general, maps of spatial turnover in genetic composition generated by GDM show similar patterns for the microsatellite and GBS datasets (Fig. 5).

**Table 3:** Results of generalized dissimilarity modeling (GDM) analysis

	Model		IBD		PC1		PC2		PC3		IBE	
	Dev.	*P*	β	*p*	β	*p*	β	*p*	β	*p*	β	*p*
SSR	**0.780**	<0.01	**0.132**	0.01	**0.465**	0.02	0.008	0.62	0.088	0.14	**0.561**	0.02
cpDNA	**0.748**	<0.01	**0.627**	<0.01	**0.442**	<0.01	0.000	0.94	0.031	0.14	**0.473**	<0.01
GBS	**0.892**	<0.01	**0.717**	<0.01	**0.537**	<0.01	0.014	0.48	0.071	0.07	**0.622**	<0.01

GDM provides a coefficient (β) for each predictor variable that estimates the contribution of that variable to explaining variation in a response variable, in this case genetic distance. The predictor variables used in our analysis included geographic distance (D) and the first three PC axes resulting from PCA analysis on 19 bioclimatic variables at each sampling site (PC1, PC2, and PC3). β_E_ represents the total contribution of environmental distance (the sum of the coefficients for each PC axis)

The overall model fit (Deviance Explained: Dev.) and significance (*p*), regression coefficients (β) and *p*-values for each predictor variable (geographic distance [IBD] and environmental PCs [PC1, PC2, and PC3]), and cumulative coefficient of IBE (for all PCs) are shown. Significant values are in bold

## Dicussion

China’s Inner Mongolia Plateau contains dramatic clines in several bioclimatic variables that are critical for plant growth and community assembly and also exhibits spatial heterogeneity in various soil properties and characteristics [49], making it an excellent landscape on which to examine the geographic and environmental drivers of population genetic structure. This region contains high elevation arid steppe and sandy soils ecosystems that are traditionally understudied in landscape genetics and phylogeography. The need to better understand population dynamics in key species in these ecosystems is pressing because the entire region is heavily threatened by soil erosion, desertification, and ongoing climate change [50].

In this study, we utilized large genomic datasets, including 22 microsatellites, cpDNA sequences from four gene regions, and 5788 SNPs, to characterize patterns of range-wide genetic differentiation across biophysical clines in *C. microphylla*, an important species for ecological restoration efforts, on the Inner Mongolia Plateau. We found that genetic diversity (haplotype diversity, nucleotide diversity, average number of alleles, and heterozygosity) was distributed across the range of the species, with more centrally located populations typically showing higher levels of genetic diversity than populations nearer to range edges, particularly to the northern range edge of this species (Table 1 and Fig. 1). This spatial pattern of genetic diversity is fairly common in many different and diverse taxa [5]. While genetic variation can have many other spatial distributions, this “abundant center hypothesis,” in which central populations harbor greater diversity appears to be one scenario that is observed fairly frequently [51, 52]. Under any scenario, better understanding the spatial distribution of genetic variation is central to developing improved management plans for maintaining genetic diversity and for predicting the impacts of potential threats to genetic diversity [52].

The exceptions to this general pattern in our study system were found in the southern populations that we sampled. Several of these populations (e.g. DL, SZQ, XH, and ZXB) harbored among the highest levels of haplotype and nucleotide diversity (based on our cpDNA dataset), allelic diversity (based on our microsatellite dataset), and observed heterozygosity (based on our microsatellite and GBS datasets; Table 1 and Fig. 1). This pattern may be explained by the historical distribution of *C. microphylla*. When the ecological niche model that we constructed was projected onto past climate layers from the last glacial maximum (LGM), it suggested that suitable habitat for *C. microphylla* was much more limited, compared to present day, and was primarily restricted to an area in the southwest corner of *C. microphylla*’s current range. This suggests that a northward expansion following glacial retreat after the LGM allowed *C. microphylla* to achieve the distribution it has today. Glacial refugia often harbor greater genetic diversity, particularly in plants [53–55], and that appears to be the case here as well, demonstrating how biogeographic histories can influence patterns of genetic diversity observed today.

To investigate how contemporary landscape factors affect patterns of genetic variation in *C. microphylla*, we conducted a landscape genetic analysis in which we employed complimentary linear and non-linear matrix regression analyses to quantify patterns of IBD and IBE. Multiple matrix regression with randomization (MMRR) [44] and generalized dissimilarity modelling (GDM) [47] revealed strong evidence that both geographic and environmental factors play important roles in structuring genetic variation in this system. Overall, the results of our MMRR and GDM analyses were highly concordant, but there were some minor differences (Tables 2, 3). Some of these differences are likely due to the linear vs. non-linear regressions used in MMRR and GDM, respectively [44, 46]. Whether the observed differences result from GDM over-fitting the model or MMRR under-fitting the model is not currently known. In any case, our results can be viewed as strong evidence that IBD and IBE are both significant patterns of genetic differentiation in *C. microphylla*, even if their precise strengths are uncertain, and therefore geographic and environmental factors are both important contributors to the genetic structuring of populations in this system.

There were also slight differences among our three genetic datasets. If we average the results for the MMRR and GDM analyses, we find that IBE played a considerably stronger role than IBD in structuring genetic variation in the microsatellite data (β_IBE_ = 0.196 vs. β_IBD_ = 0.631), but IBE and IBD were much more balanced in the cpDNA (β_IBE_ = 0.494 vs. β_IBD_ = 0.494) and GBS datasets (β_IBE_ = 0.581 vs β_IBD_ = 0.593). There is no clear reason why microsatellite markers would be expected to have lower IBD than cpDNA or GBS markers. There is an interesting possibility that chloroplast DNA, which is uni-parentally inherited through seeds, could show a different spatial pattern from nuclear DNA, which is bi-parentally inherited through seeds and pollen, if dispersal in seeds and pollen are subject to different controls. For instance, if different animals disperse seeds and pollen, which is commonly the case, or if pollen is wind dispersed while seeds fall onto the underlying terrain, then we would expect that patterns of spatial genetic variation in cpDNA and nuclear DNA could be very different [56]. For *C. microphylla*, which has pollen dispersed by wind and insect pollinators [57], we would expect that nDNA markers would show a greater signal of IBE compared to IBD, because geographic factors would be much more limiting for seed dispersal. In fact, we do see that IBE is slightly higher than IBD for our nuclear microsatellite and GBS datasets, while IBE and IBD are very similar in our cpDNA dataset, but this difference is fairly subtle.

The GDM and MMRR analyses both detected a significant signal that variation between populations in environmental PC1 drives the pattern of IBE. This signal was highly significant (*p* ≤ 0.02) and explained a large proportion of genetic variation (β = 0.383 to 0.565) in all three of our genetic datasets in the results of both MMRR and GDM analysis (Tables 2, 3). Signals for PC2 and PC3 were not significant in any dataset under either analysis. PC1 captured variation in the bioclimatic temperature variables in our environmental GIS dataset. Hence, our results suggest that IBE in *C. microphylla* is primarily driven by differences in temperature variables between populations. Both phenology and leaf emergence are linked to temperature cues in *C. microphylla* [57, 58]. Flowering period in *C. microphylla* lasts less than 30 days, and shifts in flowering period occur under different experimental temperature treatments [57]. This suggests that differences in temperature regimes between populations may cause differences in phenology which lead to reduced overlap in flowering period and, therefore, reduced gene flow. This pattern of reduced overlap in reproductive timing has been referred to as ‘isolation by time’ [59] and may be a key factor driving genetic structure in this system.

## Conclusion

Thus, overall, our study presents a strong case for a role of both historic and contemporary factors, including both geographic and environmental variables, in generating the currently observed patterns of spatial genetic variation in *C. microphylla*. For plants involved in ecological restoration, like *C. microphylla*, understanding these patterns is critical, because restoration work inherently involves moving plants between areas (even if they are geographically close). For instance, in this system, populations exhibit genetic differentiation between environments with different temperature regimes, and therefore restoration efforts should focus on transplanting between areas with similar environmental conditions. Plants adapted to different thermal climates may flower at the wrong time or out of sync with the local population, reducing the effectiveness of transplant efforts. Because *C. microphylla* also show geographically driven genetic differentiation, plants that are transplanted from distant sites that are environmentally similar could be effective at introducing genetic variation into struggling populations [50]. Hence, better understanding the factors that shape genetic variation in these species is critical for preventing unintended consequences of reintroductions and translocations and for guiding conservation plans to produce the best possible outcomes. Specifically, the knowledge generated by studies like ours can contribute to managing how genetic variation is distributed in these species, the probability of individuals surviving and becoming established under various climatic factors, and the chances of maintaining genetically healthy populations.

## Additional file

Additional file 1: Table S1. Sampled populations, geographical variables and individual numbers used in the study for different molecular marker datasets. **Table S2.** The 22 SSR loci analyzed in our 10 studied *C. microphylla* populations. **Table S3.** 19 bioclimatic variables of 10 *C. micraphylla* populations. **Table S4.** Pairwise F_ST_ detected by SSRs among our 10 studied *C. microphylla* populations. **Table S5.** Pairwise F_ST_ detected by cpDNA among our 10 studied *C. microphylla* populations. **Table S6.** Pairwise F_ST_ detected by GBS among 9 of our studied *C. microphylla* populations. **Figure S1.** Plots of our 10 sample localities in climate space. Each point represents the environmental values of a population on PC axes 1 and 2 (left) and PC axes 2 and 3 (right). The distribution of points across the climate space graphs shows how our sampling scheme captures a wide range of environmental variation. **Figure S2.** Population genetic structure analysis for SSR (A) and GBS (B) based markers of *C. microphylla*, showing the ΔK statistics calculated according to Evanno et al. (2005). **Figure S3.** Jackknife analyses of individual predictor importance for *C. microphylla* applied to the Maxent model, presented in relation to overall model quality or “total gain”. Dark blue bars indicate the gain achieved when including that predictor only and excluding remaining predictors; light blue bars show how the total gain is diminished without the given predictor. (DOCX 219 kb)

## Abbreviations

ENM: Ecological niche modelling
GBS: Genotyping-by-sequencing
GDM: Generalized dissimilarity modelling
IBD: Isolation-by-distance
IBE: Isolation-by-environment
LGM: Last Glacial Maximum
MMRR: Multiple matrix regression with randomization
SSR: Simple sequence repeat
