# Supplementary material for: Population genetic structure is shaped by historical, geographic, and environmental factors in the leguminous shrub Caragana microphylla on the Inner Mongolia Plateau of China
Source: BMC Plant Biol. 2017 Nov 13;17:200. doi: 10.1186/s12870-017-1147-7 (PMC5683519; doi:10.1186/s12870-017-1147-7)
Supplement: Additional file 1: Table S1. — Sampled populations, geographical variables and individual numbers used in the study for different molecular marker datasets. Table S2. The 22 SSR loci analyzed in our 10 studied C. microphylla populations. Table S3. 19 bioclimatic variables of 10 C. micraphylla populations. Table S4. Pairwise FST detected by SSRs among our 10 studied C. microphylla populations. Table S5. Pairwise FST detected by cpDNA among our 10 studied C. microphylla populations. Table S6. Pairwise FST detected by GBS among 9 of our studied C. microphylla populations. Figure S1. Plots of our 10 sample localities in climate space. Each point represents the environmental values of a population on PC axes 1 and 2 (left) and PC axes 2 and 3 (right). The distribution of points across the climate space graphs shows how our sampling scheme captures a wide range of environmental variation. Figure S2. Population genetic structure analysis for SSR (A) and GBS (B) based markers of C. microphylla, showing the ΔK statistics calculated according to Evanno et al. (2005). Figure S3. Jackknife analyses of individual predictor importance for C. microphylla applied to the Maxent model, presented in relation to overall model quality or “total gain”. Dark blue bars indicate the gain achieved when including that predictor only and excluding remaining predictors; light blue bars show how the total gain is diminished without the given predictor. (DOCX 219 kb) [file 12870_2017_1147_MOESM1_ESM.docx]

**Table S1.** Sampled populations, geographical variables and individual number used in the study for different marker system

| **Population** | **Location** | **Latitude(°N)** | **Longitude(°E)** | | **Terrain** | | | **Soil** | | | **N_SSR_** | **N_cpDNA_** | **N_GBS_** |
| --- | --- | --- | --- | --- | --- | --- | --- | --- | --- | --- | --- | --- | --- |
| SZW | Siziwang | 42°05'47″ | 111°54'49″ | | Hill | | | NA | | | 21 | 21 | 21 |
| ZXB | Zhengxiangbai | 42°36'39″ | 115°12'33″ | | Hill | | | Chestnut soil | | | 24 | 24 | 7 |
| DL | Duolun | 42°15'14″ | 116°13'40″ | | Basin, shady slope | | | NA | | | 22 | 22 | 15 |
| XH | Xianghuang | 42°12'11″ | 113°50'41″ | | High plain | | | NA | | | 24 | 24 | 8 |
| QYH | Qahar Youyi Hou | 41°27'49″ | 113°3'5″ | | Hill | | | NA | | | 23 | 23 | 18 |
| XU | Xi Ujimqin | 44°30'1″ | 117°3'5″ | | Fixed dune | | | NA | | | 22 | 22 | 12 |
| DU | Dong Ujimqin | 45°16'9″ | 116°4'44″ | | Hill, sunny slope | | | Chernozem | | | 18 | 18 | 6 |
| EWK | Ewenki | 49°7′28″ | 120°7′36″ | | Sandy land | | | Sandy soil | | | 22 | 22 | 10 |
| CB | ChenBarag | 49°8′55″ | 118°51′38″ | | Sandy land | | | Sandy soil | | | 24 | 24 | 10 |
| XBY | Xin Barag you | 48°48′25″ | 116°59′48″ | | Lakeside | | | Sandy soil | | | 21 | 21 | 20 |
| Total |  |  |  | |  | | |  | | | 221 | 221 | 127 |

NA = Not available

**Table S2.** The 22 SSR loci analyzed in 10 *C. microphylla* populations.

| **Markers** | **Genebank accession** | **Forward primer (5'-3')** | **Reverse primer (5'-3')** | **Expected size (bp)** |
| --- | --- | --- | --- | --- |
| CM01 | KC959482 | TGATTAACAAAGATGTGTTGCCTT | ATATTGCCCTCCTTCCTTGG | 242 |
| CM02 | KC959488 | CCCGAAACAAACAAAAGGAA | GTACGTACGGGAAAGGGTGA | 278 |
| CM04 | KC959490 | CCCAAAACAAACAAAGGGAA | GTACGTACGGGAAAGGGTGA | 269 |
| CM05 | KC959491 | GGAGACTTCACCAGTGCCAT | ACCCCTAGGCTGACCTGACT | 187 |
| CM06 | KC959491 | CCCAGTGTTTCTCACCGTTT | CTTCACCGATTCCAACTTCG | 195 |
| CM07 | KC959493 | CAAAAACCAACCCAAAACAAA | GTACGTACGGGAAAGGGTGA | 280 |
| CM11 | KC959498 | ATTGAAACCATTTGGATCGG | ACACCACCACAACCCATTTT | 236 |
| CM13 | KC959501 | TCACTTGATATGATCTCCCCC | GTCCCTGTGCAAATTGTTCC | 203 |
| CM18 | KC959507 | ATTGAAACCATTTGGATCGG | ACACCACCACAACCCATTTT | 236 |
| CM21 | KC959511 | CAGACGAGGATTTGCTGC | AGTGAAAGTGGGTGTGGAGG | 197 |
| CM22 | KC959511 | GTTGACTGCACGAATGTTGG | ATGCGTGTGAAGGTGAGAGA | 183 |
| CM23 | KC959512 | CACGAAAGGCTACAACTTTGG | ACCACCGCTCTTTAATTCCC | 270 |
| CM27 | KC959516 | TTCCCAAACCGAAGAAACAC | ATTGCGGGACATCAAATAGG | 251 |
| CM31 | KC959521 | TTCCCAAACCGAAGAAACAC | ATTGCGGGACATCAAATAGG | 245 |
| CM37 | KC959529 | TTATCACACGGGCATGACTC | CTAAACCCGAACCAAACCAA | 266 |
| CM39 | KC959531 | AAGCAGATCCAGAAGTGGCA | ACAATGGGACAGTGGGTCTC | 209 |
| CM42 | KC959533 | ACAATGGGACAGTGGGTCTC | TGCTGACAGATGATCTAGGGC | 239 |
| CM44 | KC959535 | AAAAGGGAGTGTGGTTGTGG | TGCTGACAGATGATCTAGGGC | 210 |
| CM45 | KC959536 | TGCTGACAGATGATCTAGGGC | ACAATGGGACAGTGGGTCTC | 239 |
| CM49 | KC959540 | TGCTGACAGATGATCTAGGGC | ACAATGGGACAGTGGGTCTC | 238 |
| CM52 | KC959542 | TGCTGACAGATGATCTAGGGC | ACAATGGGACAGTGGGTCTC | 240 |
| CM54 | KC959543 | ACAATGGGACAGTGGGTCTC | TGCTGACAGATGATCTAGGGC | 242 |

**Table S3.** 19 bioclimatic variables for 10 *C. microphylla* populations.

| Pop | Bio1 | Bio2 | Bio3 | Bio4 | Bio5 | Bio6 | Bio7 | Bio8 | Bio9 | Bio10 | Bio11 | Bio12 | Bio13 | Bio14 | Bio15 | Bio16 | Bio17 | Bio18 | Bio19 |
| --- | --- | --- | --- | --- | --- | --- | --- | --- | --- | --- | --- | --- | --- | --- | --- | --- | --- | --- | --- |
| SZW | 31 | 131 | 26 | 13237 | 269 | -230 | 499 | 193 | -150 | 193 | -150 | 211 | 65 | 2 | 110 | 140 | 6 | 140 | 6 |
| ZXB | 27 | 126 | 25 | 13315 | 259 | -232 | 491 | 187 | -156 | 187 | -156 | 310 | 86 | 2 | 106 | 207 | 8 | 207 | 8 |
| DL | 14 | 126 | 26 | 12891 | 238 | -241 | 479 | 168 | -164 | 168 | -164 | 376 | 109 | 2 | 106 | 251 | 9 | 251 | 9 |
| XH | 33 | 118 | 25 | 12557 | 256 | -210 | 466 | 186 | -138 | 186 | -138 | 294 | 78 | 2 | 106 | 200 | 7 | 200 | 7 |
| QYH | 34 | 128 | 27 | 11927 | 254 | -205 | 459 | 180 | -128 | 180 | -128 | 329 | 91 | 2 | 107 | 223 | 8 | 223 | 8 |
| XU | 13 | 135 | 26 | 13876 | 260 | -255 | 515 | 182 | -176 | 182 | -176 | 317 | 92 | 3 | 108 | 218 | 9 | 218 | 9 |
| DU | 9 | 137 | 25 | 14316 | 266 | -262 | 528 | 184 | -185 | 184 | -185 | 257 | 72 | 2 | 109 | 178 | 6 | 178 | 6 |
| EWK | -11 | 128 | 22 | 15531 | 254 | -304 | 558 | 177 | -189 | 177 | -224 | 379 | 107 | 3 | 110 | 262 | 12 | 262 | 12 |
| CB | -9 | 131 | 22 | 16053 | 265 | -310 | 575 | 185 | -194 | 185 | -228 | 328 | 101 | 3 | 114 | 232 | 10 | 232 | 10 |
| XBY | 6 | 127 | 23 | 15331 | 271 | -274 | 545 | 195 | -167 | 195 | -200 | 262 | 87 | 1 | 127 | 196 | 4 | 196 | 4 |

Bio1 = Annual Mean Temperature, Bio2 = Mean Diurnal Range (Mean of monthly (max temp - min temp)), Bio3 = Isothermality (P2/P7) (* 100), Bio4 = Temperature Seasonality (standard deviation *100), Bio5 = Max Temperature of Warmest Month, Bio6 = Min Temperature of Coldest Month, Bio7 = Temperature Annual Range (P5-P6), Bio8 = Mean Temperature of Wettest Quarter, Bio9 = Mean Temperature of Driest Quarter, Bio10 = Mean Temperature of Warmest Quarter, Bio11 = Mean Temperature of Coldest Quarter, Bio12 = Annual Precipitation, Bio13 = Precipitation of Wettest Month, Bio14 = Precipitation of Driest Month, Bio15 = Precipitation Seasonality (Coefficient of Variation), Bio16 = Precipitation of Wettest Quarter, Bio17 = Precipitation of Driest Quarter, Bio18 = Precipitation of Warmest Quarter, Bio19 = Precipitation of Coldest Quarter.

**Table S4.** Pairwise F_ST_ detected by SSRs among 10 *C. microphylla* populations.

|  | **SZW** | **ZXB** | **DL** | **XH** | **QYH** | **XU** | **DU** | **EWK** | **CB** | **XBY** |
| --- | --- | --- | --- | --- | --- | --- | --- | --- | --- | --- |
| SZW | 0.000 |  |  |  |  |  |  |  |  |  |
| ZXB | 0.019* | 0.000 |  |  |  |  |  |  |  |  |
| DL | 0.029** | 0.017* | 0.000 |  |  |  |  |  |  |  |
| XH | 0.017* | 0.011^ns^ | 0.019* | 0.000 | 0.000 |  |  |  |  |  |
| QYH | 0.039** | 0.051** | 0.070** | 0.035** | 0.000 |  |  |  |  |  |
| XU | 0.125** | 0.131** | 0.143** | 0.133** | 0.123** | 0.000 |  |  |  |  |
| DU | 0.047** | 0.048** | 0.062** | 0.046** | 0.056** | 0.049** | 0.000 |  |  |  |
| EWK | 0.157** | 0.160** | 0.164** | 0.159** | 0.167** | 0.103** | 0.101** | 0.000 |  |  |
| CB | 0.195** | 0.200** | 0.206** | 0.199** | 0.209** | 0.124** | 0.144** | 0.092** | 0.000 | 0.000 |
| XBY | 0.183** | 0.179** | 0.196** | 0.199** | 0.221** | 0.124** | 0.152** | 0.139** | 0.145** | 0.000 |

^**^, significance at P < 0.01, ^*^, significance at P < 0.05, ^ns^ significance at P > 0.05

**Table S5.** Pairwise F_ST_ detected by cpDNA among 10 *C. microphylla* populations.

|  | **SZW** | **ZXB** | **DL** | **XH** | **QYH** | **XU** | **DU** | **EWK** | **CB** | **XBY** |
| --- | --- | --- | --- | --- | --- | --- | --- | --- | --- | --- |
| SZW | 0.00 |  |  |  |  |  |  |  |  |  |
| ZXB | 0.13** | 0.00 |  |  |  |  |  |  |  |  |
| DL | 0.11** | 0.08* | 0.00 |  |  |  |  |  |  |  |
| XH | 0.06 ^ns^ | 0.03 ^ns^ | 0.11* | 0.00 |  |  |  |  |  |  |
| QYH | 0.03 ^ns^ | 0.13** | 0.13** | 0.00 ^ns^ | 0.00 |  |  |  |  |  |
| XU | 0.44** | 0.52** | 0.38** | 0.51** | 0.50** | 0.00 |  |  |  |  |
| DU | 0.46** | 0.55** | 0.38** | 0.54** | 0.49** | 0.07 | 0.00 |  |  |  |
| EWK | 0.62** | 0.71** | 0.58** | 0.69** | 0.65** | 0.19* | 0.14 ^ns^ | 0.00 |  |  |
| CB | 0.63** | 0.71** | 0.59** | 0.50** | 0.66** | 0.20* | 0.15 ^ns^ | 0.00 | 0.00 |  |
| XBY | 0.61** | 0.69** | 0.57** | 0.69** | 0.64** | 0.19* | 0.13 ^ns^ | 0.00 | 0.00 | 0.00 |

^**^, significance at P < 0.01, ^*^, significance at P < 0.05, ^ns^ significance at P > 0.05

**Table S6.** Pairwise F_ST_ detected by GBS among 9 *C. microphylla* populations.

|  | **ZXB** | **DL** | **XH** | **QYH** | **XU** | **DU** | **EWK** | **CB** | **XBY** |
| --- | --- | --- | --- | --- | --- | --- | --- | --- | --- |
| ZXB | 0.000 |  |  |  |  |  |  |  |  |
| DL | 0.029^ns^ | 0.000 |  |  |  |  |  |  |  |
| XH | 0.073^*^ | 0.049 ^ns^ | 0.000 |  |  |  |  |  |  |
| QYH | 0.066^*^ | 0.083^*^ | 0.024 ^ns^ | 0.000 |  |  |  |  |  |
| XU | 0.182^**^ | 0.139^**^ | 0.209^**^ | 0.228^**^ | 0.000 |  |  |  |  |
| DU | 0.135^**^ | 0.085^*^ | 0.167^**^ | 0.170^**^ | 0.056^*^ | 0.000 |  |  |  |
| EWK | 0.297^**^ | 0.234^**^ | 0.349^**^ | 0.366^**^ | 0.125^**^ | 0.138^**^ | 0.000 |  |  |
| CB | 0.335^**^ | 0.263^**^ | 0.385^**^ | 0.398^**^ | 0.151^**^ | 0.170^**^ | 0.063^*^ | 0.000 |  |
| XBY | 0.383^**^ | 0.336^**^ | 0.420^**^ | 0.431^**^ | 0.204^**^ | 0.221^**^ | 0.126^**^ | 0.143^**^ | 0.000 |

^**^, significance at P < 0.01, ^*^, significance at P < 0.05, ^ns^ significance at P > 0.05

**Fig. S1.** Plots of our 10 sample localities in climate space. Each point represents the environmental values of a population on PC axes 1 and 2 (left) and PC axes 2 and 3 (right). The distribution of points across the climate space graphs shows how our sampling scheme captures a wide range of environmental variation.

**
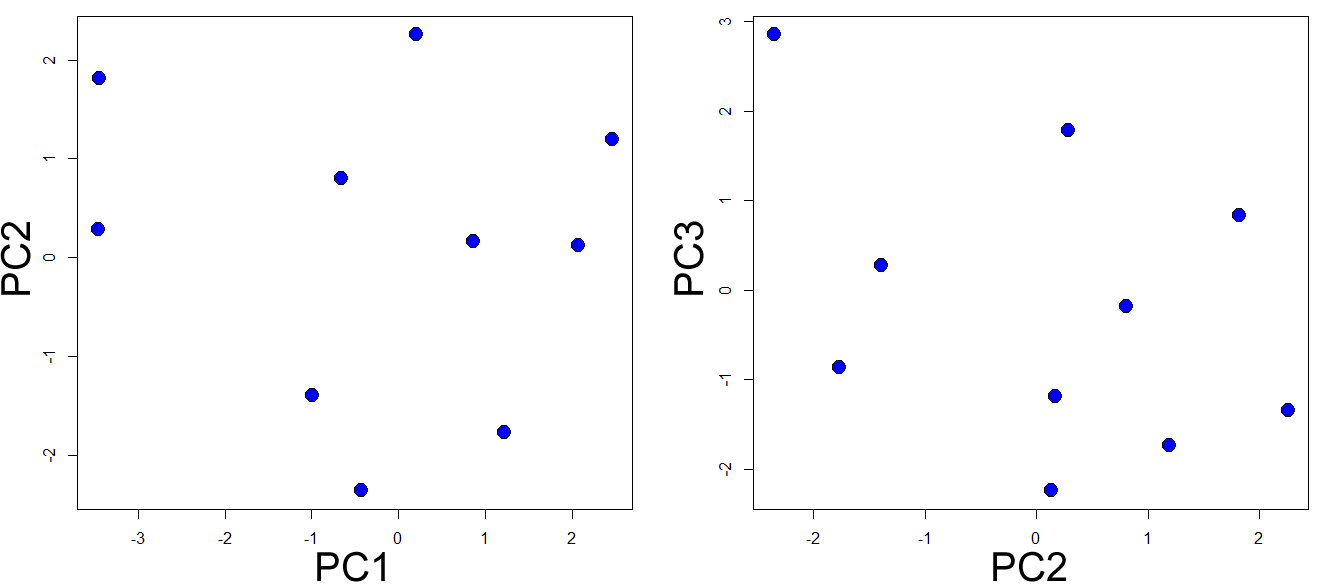
**

**Fig. S2**. Population genetic structure analysis for SSR (A) and GBS (B) based markers of *C. microphylla*, showing the ΔK statistics calculated according to Evanno et al. (2005).


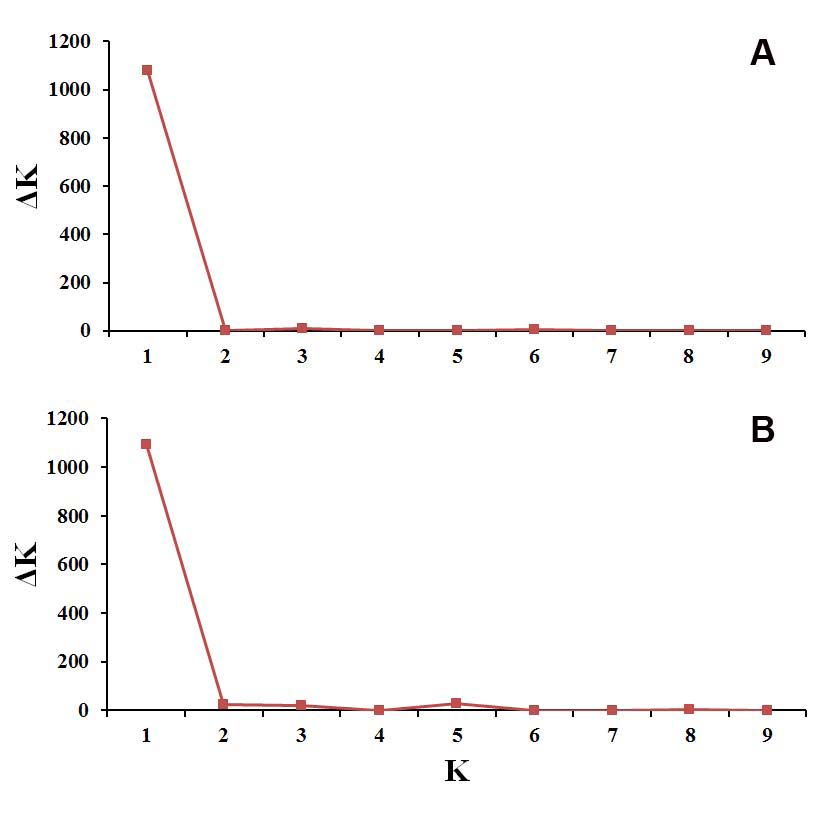


**Fig. S3.** Jackknife analyses of individual predictor importance for *C. microphylla* applied to the Maxent model, presented in relation to overall model quality or “total gain”. Dark blue bars indicate the gain achieved when including that predictor only and excluding remaining predictors; light blue bars show how the total gain is diminished without the given predictor.

**
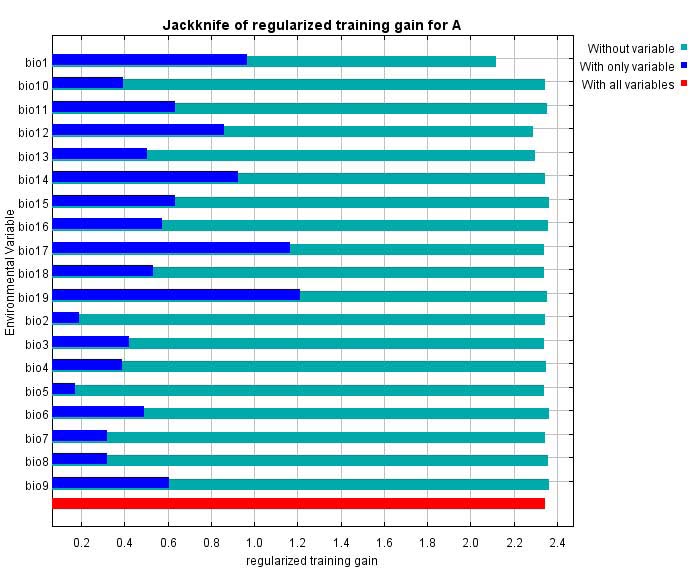
**
